# Adaptively coupled phase retrieval in multi-peak Bragg coherent diffraction imaging

**DOI:** 10.1107/S1600576725010131

**Published:** 2026-02-01

**Authors:** J. Nicholas Porter, Yueheng Zhang, Ross J. Harder, Barbara Frosik, Wonsuk Cha, Yuan Gao, Garth Williams, Joshua Miller, Nash Karrington, Andres Herrera, Anthony Rollett, Stephan Hruszkewycz, Richard L. Sandberg

**Affiliations:** a Department of Physics and Astronomy Brigham Young University,Provo UT 84602 USA; b Department of Materials Science and Engineering, Carnegie Mellon University, Pittsburgh, PA 15213, USA; c Advanced Photon Source, Argonne National Laboratory, Lemont, IL 60439, USA; d National Synchrotron Light Source II Brookhaven National Laboratory,Upton NY 11973 USA; e Materials Science Division, Argonne National Laboratory, Lemont, IL 60439, USA; Brazilian Synchrotron Light Laboratory, Brazil

**Keywords:** X-ray diffraction, strain microscopy, Bragg coherent diffraction imaging, phase retrieval

## Abstract

Recent advances in Bragg coherent diffraction imaging (BCDI) experimental techniques permit routine measurement of multiple Bragg peaks from a single crystalline grain. The resulting images contain the full lattice distortion vector field which can be differentiated to provide lattice strain and rotation. With the advent of fourth-generation synchrotron light sources, such multi-peak datasets are produced at high rates, facilitating the need for rapid phase retrieval of the multiple peaks and subsequent image analysis. Here we describe and demonstrate a new implementation of a coupled phase retrieval technique for multi-peak BCDI which simultaneously treats each Bragg peak of the dataset and produces a three-dimensional image of the crystal’s morphology and lattice distortion field. In addition, this method uses the redundant information contained in the various Bragg diffraction patterns to detect and suppress spurious signal appearing on the detector in a subset of the measurements. Compared with manual data editing, adaptive coupling produces a more consistent phase profile in reciprocal space and sharper surfaces in direct space, with no significant difference in computational cost. These improvements reduce the need for manual preprocessing and enable robust high-throughput analysis of multi-peak BCDI data, supporting near-real-time strain microscopy at modern synchrotron facilities.

## Abstract 2

We present an adaptively coupled phase retrieval algorithm for multi-peak Bragg coherent diffraction imaging. The technique uses the redundant information contained in separate Bragg peaks to detect and remove spurious signal.

## 1. Introduction

Bragg coherent diffraction imaging (BCDI) is an important form of lensless imaging (Williams *et al.*, 2003 ▸; Pfeifer *et al.*, 2006 ▸; Newton *et al.*, 2010 ▸; Miao *et al.*, 2015 ▸). Like other forms of coherent diffraction imaging, it is based on the retrieval of phase information from spatially oversampled coherent diffraction intensities (Fienup, 1982 ▸; Fienup, 1987 ▸; Marchesini *et al.*, 2003 ▸), which can then be back-propagated to create a nanometre-scale-resolution image of the diffracting object once phase retrieval is complete. In the case of BCDI, this diffraction pattern is obtained by reflecting coherent X-rays from atomic planes within a crystal according to Bragg’s law (Patterson, 1939 ▸). In this geometry, ‘rocking’ the crystal through the Bragg condition in fine angular increments while measuring the two-dimensional diffraction intensity around a Bragg peak is equivalent to sweeping out a three-dimensional volume of that crystal’s reciprocal space (Williams *et al.*, 2003 ▸). In the elastic single scattering limit, this measurement is mathematically related to the object through a single 3D Fourier transform operation.

Bragg diffraction is highly sensitive to the spacing of atomic planes, and any variation in that spacing due to crystalline deviatoric strain is encoded in the coherent interference surrounding the corresponding Bragg peak. For a small region around the reciprocal-lattice point **G**, this encoding can be approximated as 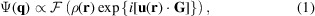
where Ψ is the complex diffraction pattern, ρ is proportional to the diffracting crystal’s electron density and **u** is the atomic displacement field. Because of the projection **u** · **G**, at least three noncoplanar reflections are required to provide a full 3D image of the lattice distortion (and by extension the full strain tensor) within a crystal (Pfeifer *et al.*, 2006 ▸; Harder *et al.*, 2007 ▸; Robinson & Harder, 2009 ▸; Favre-Nicolin *et al.*, 2010 ▸; Newton *et al.*, 2010 ▸; Yang *et al.*, 2013 ▸; Ulvestad *et al.*, 2016 ▸; Hofmann *et al.*, 2017 ▸; Ulvestad *et al.*, 2017 ▸; Yau *et al.*, 2017 ▸; Cherukara *et al.*, 2018 ▸; Hruszkewycz *et al.*, 2018 ▸; Singer *et al.*, 2018 ▸; Kawaguchi *et al.*, 2019 ▸; Hofmann *et al.*, 2020 ▸).

In recent years, several coupled phase retrieval (CPR) techniques have been demonstrated in which, rather than combining the results of individually phased Bragg peaks, the phase retrieval problems associated with each peak are synthesized into a single optimization problem (Newton, 2020 ▸; Gao *et al.*, 2021 ▸; Wilkin *et al.*, 2021 ▸; Maddali *et al.*, 2023 ▸). This larger problem requires a reconstructed object to agree simultaneously with all Bragg peaks. Due to the limitations of measurement, a low-error solution to the phase problem that satisfies all collected data is pathologically rare. However, for small amounts of evenly distributed noise, an equal compromise between all measurements is likely to be closer to the truth than a perfect fit to any one.

**Figure 1:**
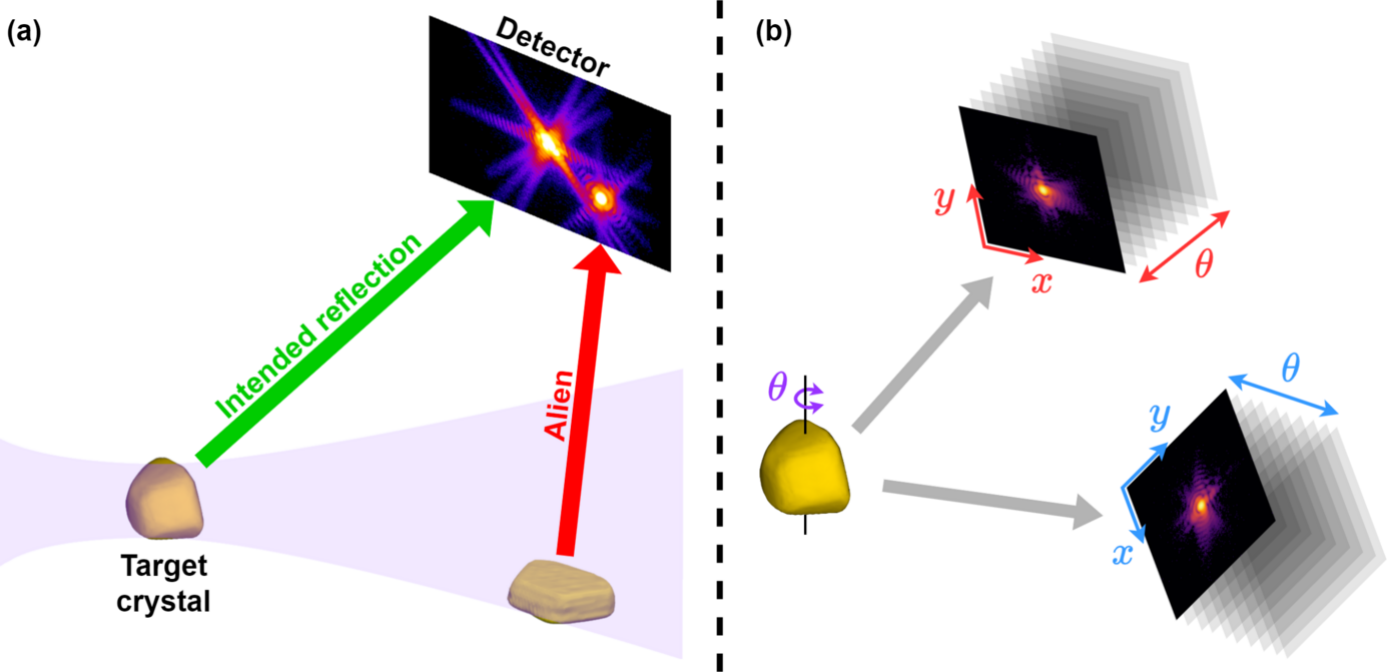
Illustrations of two significant challenges to multi-peak BCDI. (*a*) An alien is produced when a single experimental geometry satisfies the Bragg condition for multiple crystals along the beam path, preventing either peak from being faithfully measured. (*b*) Each peak is measured with a unique crystal orientation and detector position, giving each its own unique reciprocal basis.

Unfortunately, the equal-compromise approach – which we will refer to as static coupling – fails in the case of a large localized discrepancy, such as a second Bragg peak caused by another (unwanted) crystal, as shown in Fig. 1 ▸(*a*). These accidental reflections are colloquially referred to as ‘aliens’, a term comparing their unexpected appearance and unknown origin to visitors from outer space. The generally preferred approach to the alien problem has been to remove the problematic data manually, either by erasing sections of a measured Bragg peak or by discarding the measurement entirely. However, while increasingly brilliant X-ray facilities worldwide allow for more sensitive measurements, that very sensitivity will also reduce the amount of data that can be collected without aliens. This, combined with an ever-accelerating rate of data production, necessitates a more efficient, precise and objective method for alien removal.

A second challenge associated with multi-peak BCDI, illustrated in Fig. 1 ▸(*b*), is that the coordinate system used for measurement is different for each peak (Maddali *et al.*, 2020 ▸). One solution is to resample the data into a common reference frame before performing phase retrieval. However, transforming the unique geometry of each peak measurement to a common frame skews the initially rectangular 3D images into parallelepipeds which only partially fill the array onto which they are resampled. As a result, diffraction bars which extended to the edges of the original measurement may be truncated in the resampled array, producing Gibbs-phenomenon artifacts in the reconstruction. The alternative, transforming the reconstructing object into each measurement geometry when phasing the corresponding peak, has its own drawbacks. While often preferred for being more faithful to the measurement, this method can require thousands of interpolations throughout a reconstruction, which introduces a risk of compounding errors and increases the reconstruction time by orders of magnitude.

In this article, we present two adaptive coupling methods for multi-peak BCDI that use redundant information to mitigate the effects of spurious or incomplete measurements. These methods vastly increase the robustness of CPR, producing high-quality reconstructions despite aliens and resampled data, all while adding a negligible computational cost. By reducing the need for manual data processing and enabling faster reconstructions, adaptive coupling can dramatically accelerate the analysis of multi-peak BCDI data. Section 2 describes both techniques in detail, including how they fit into a particular CPR framework. In Section 3 we demonstrate these methods by reconstructing the density and atomic displacement field of an Au crystal from five Bragg peaks, one of which contains an alien. Finally, Section 4 concludes the article with a brief discussion of the potential implications of such a technique. Table 1 ▸ lists the symbols and notation used herein.

## 2. Adaptive coupling

The adaptive coupling methods presented here are implemented within a CPR framework that involves phasing a single peak at a time, while periodically applying a peak-switching operation. In this process, the redundant information gained while phasing one peak generally improves the initial guess for the next peak. Each peak *p* has an associated scattering vector **G**_*p*_, which determines the diffraction measurement 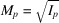 used during phasing. When switching from a given peak, the partially reconstructed exit wave ψ(**r**) is first used to update a shared density ρ(**r**) and displacement field **u**(**r**) as follows: 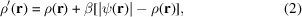

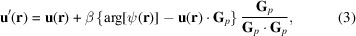
where β is a weighting parameter which biases the update towards new (β = 1) or old (β = 0) information and may be adjusted at any point in the reconstruction. The updated density/displacement are then projected onto the new peak using equation (1) and phasing continues with the corresponding diffraction pattern. This static coupling largely follows the CPR method described by Gao *et al.* (2021 ▸), the main differences being the weighting parameter β and the option to allow multiple iterations between peak switches.

**Figure 2:**
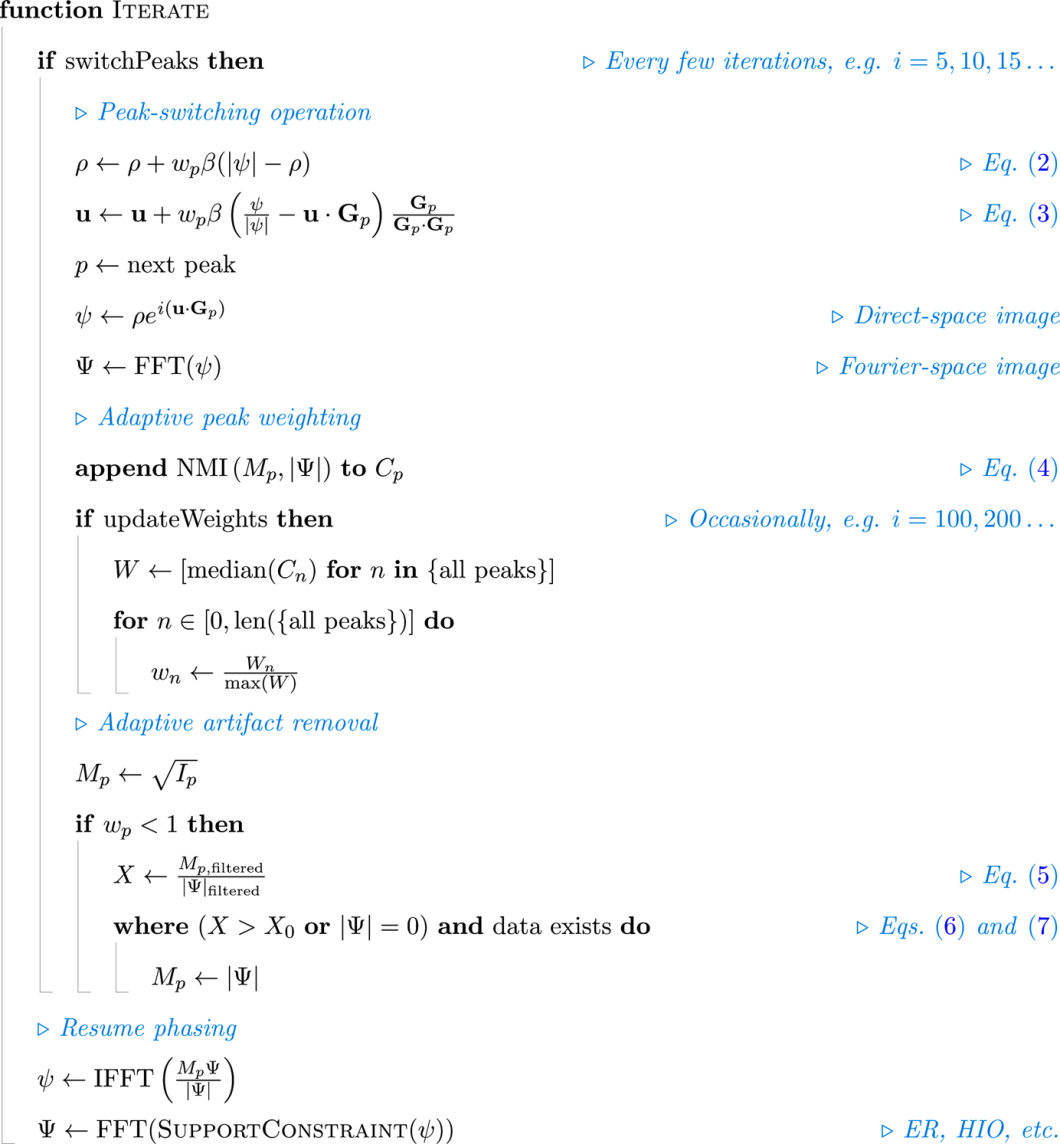
A pseudocode representation of a single iteration of adaptively coupled phase retrieval. The Boolean values of switchPeaks and updateWeights depend on whether the current iteration has been marked for those operations. The FFT and IFFT functions, respectively, represent the forward and inverse fast Fourier transforms.

Into this framework we implement two novel techniques: adaptive peak weighting and adaptive artifact removal. A single iteration of the complete algorithm is given in Fig. 2 ▸. On a conceptual level, both methods work on the principle that, since each Bragg peak is a projection of the same crystal, their fully described wavefields must all be perfectly compatible – any conflicting information represents an error in measurement. As such, adaptive coupling is concerned with determining the compatibility of measurements and weighting them accordingly. In the following descriptions, Ψ refers to the diffraction pattern as projected from the density and displace­ment fields [see equation (1)] without any additional phasing. For simplicity, we shall assume that the various Bragg peaks have been resampled in a common basis.

Adaptive peak weighting regulates the overall influence each dataset has on the reconstruction. As a confidence metric, we calculate the normalized mutual information (NMI) defined as 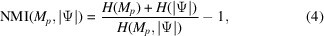
where *M*_*p*_ and |Ψ| are, respectively, the measured and calculated diffraction amplitudes, both of which have been convolved with a Gaussian (σ = 1 voxel) to suppress high-frequency fluctuations, and *H* is the numerically estimated Shannon entropy of an array (or joint entropy of two arrays). Mutual information compares the overall structure by examining the frequency with which two similar-valued elements in one array are also similar-valued in the other (Feixas *et al.*, 2014 ▸). For a more detailed discussion of NMI, see Appendix *A*[App appa]. After switching to a peak but before phasing begins, the NMI is calculated and appended to a running list *C*_*p*_ associated with that peak.

In general, the NMI should increase as the reconstruction progresses, and a persistently low NMI indicates the presence of spurious information. On this basis, each peak is periodically assigned a weight *w*_*p*_, defined as the median value of *C*_*p*_ (scaled so that the ‘heaviest’ peak has unit weight). The global weighting parameter β is then replaced with *w*_*p*_β in equations (2) and (3), reducing the update strength for all but the most confident peak. Additionally, peaks whose weight falls below a user-defined threshold *w*_0_ are not allowed to alter the support region while phasing, for example, with *Shrinkwrap* (Marchesini *et al.*, 2003 ▸).

Adaptive artifact removal applies the same high-level principle on a voxel-by-voxel basis. Aliens and other artifacts are detected using an error function, 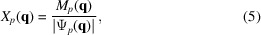
which is large where the measurement exceeds the projection but small where the projection exceeds the measurement. This asymmetry makes it well suited to detecting aliens, which are almost exclusively an additive phenomenon. In addition to aliens, any voxel with a value of zero may be considered an artifact. In cases of resampled diffraction patterns, this includes regions where the measurement does not occupy the entire resampling array. Even in the original measurements, however, zero-valued voxels do not indicate that the photon distribution integrated over the area of a pixel is equal to zero. Rather, they are a result of finite dynamic range, photon statistics and/or image processing (*e.g.* thresholding to remove the noise floor).

When switching to a new peak, we define a mask 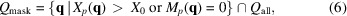
where *X*_0_ is a user-defined threshold and *Q*_all_ is the volume of reciprocal space that has been measured by at least one peak. This mask selects low-confidence voxels which could benefit from information drawn from other peaks. If the current peak has *w_p_* < 1, we redefine the reciprocal-space image used for the modulus constraint as 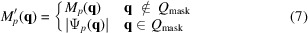
until the next peak switch. This modified modulus constraint enforces consistency both with the reliable portions of the measured diffraction pattern and with the projection synthesized from all other Bragg peaks. If the peak has *w_p_* = 1, the modulus constraint is left unchanged, to prevent any voxel from being masked in every peak and therefore unconstrained. A single projection guess, defined as the median measured amplitude at each point in reciprocal space, is used to produce an initial mask for each peak. Until the mask is updated, these voxels should be constrained to zero to prevent particularly bright aliens from becoming entrenched. Note that this assumes that, at any given spatial frequency (relative to its associated Bragg peak), the majority of measurements will not contain an alien.

In addition to the ‘recipe’ associated with nearly all iterative phase retrieval algorithms, adaptive coupling requires the user to set three parameters: (i) corresponding lists of iterations/values for updating *w*_0_, (ii) how often to update each peak’s *Q*_mask_ and (iii) the alien detection threshold *X*_0_. However, as is often the case, a single set of default parameters can be used for a wide range of datasets. As general guidelines, we recommend *w*_0_ be initially set to 0.5 and then increased periodically to a final value of 0.9; *Q*_mask_ should be updated after the solver has cycled through each peak two to five times; and *X*_0_ ∈ [2, 6] depending on the desired sensitivity.

## 3. Reconstructions

**Figure 3:**
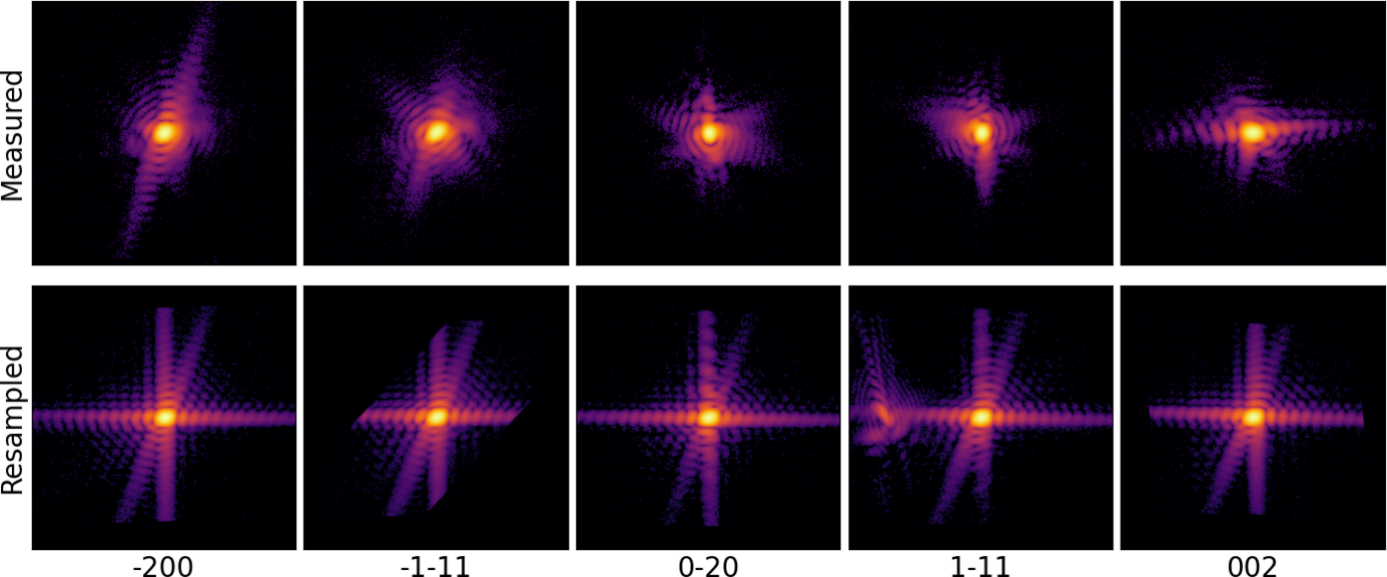
Central slices of each measured Bragg peak, (top) in the original rocking curve coordinate system and (bottom) resampled into the crystal coordinate system for phase retrieval. Images are colored on a logarithmic scale. An additional diffraction pattern was added to the 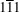 peak image to simulate the presence of an alien. Due to the placement of this alien, it does not appear in the central frame of the rocking curve, though some fringes are visible to the left of the main peak in the resampled image. The alien can be more easily seen in Fig. 6(*b*).

**Figure 4:**
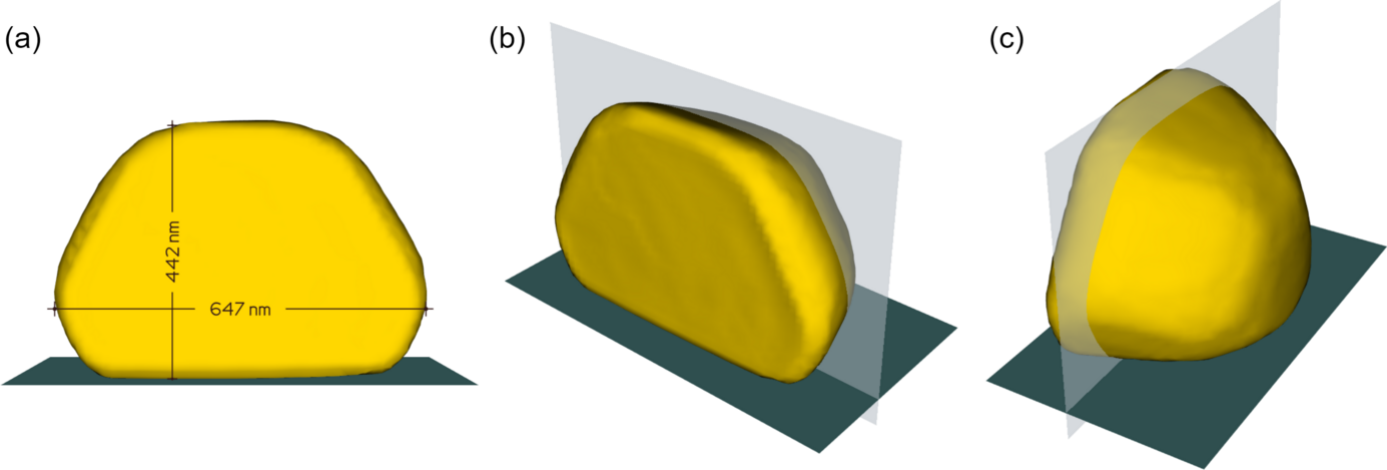
Three views of a 60% density isosurface of an Au crystal as reconstructed using adaptive coupling. The dimensions given in (*a*) represent the height and width of the crystal at its largest. The cross section used in Fig. 8 is shown as a partially transparent plane in (*b*) and (*c*). The dark-gray plane in all three images represents the substrate on which this crystal sits. The crystal’s unusual morphology is due to it being only one half of a bicrystal. The forward-facing facet in panel (*a*) is a grain boundary with a twinned crystal (not shown) of similar size and shape.

The effectiveness of adaptive coupling is demonstrated by reconstructing an Au crystal (Fig. 3 ▸) from its 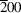, 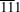, 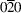, 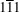 and 002 Bragg peaks. These data were collected at the Advanced Photon Source (APS) on beamline 34-ID-C in 2022, prior to the APS upgrade. Diffraction patterns were produced with a 9 keV beam and measured at a distance of 1 m on one 256 × 256 pixel quadrant of an ASI Quad Timepix detector at 200 points along a rocking curve (±0.5°). The images were resampled in the crystal’s reference frame immediately prior to reconstruction but after all other pre-processing. The resampled arrays used for phasing were 240 × 240 × 240 voxels. Central slices of both the original and resampled diffraction images are shown in Fig. 3 ▸, and the morphology of the reconstructed crystal is given in Fig. 4 ▸.

Before resampling, a second diffraction pattern was superimposed onto the 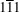 peak. This artificial alien, originally measured from a separate Au crystal, was scaled to have a peak intensity 10% that of the primary diffraction pattern, with Poisson noise regenerated to match the dynamic range. The alien was intentionally placed along one of the primary’s diffraction bars, such that erasing the alien would also erase some of the intended measurement.

Each reconstruction was performed in *Cohere*, an open-source BCDI-focused phase retrieval program developed at the APS (Frosik *et al.*, 2024 ▸), with 1000 total iterations of phase retrieval broken into alternating blocks of 75 hybrid input–output (HIO) steps and 25 error reduction (ER) steps (Fienup, 1982 ▸). *Shrinkwrap* (Marchesini *et al.*, 2003 ▸) was applied with 1 voxel Gaussian pre-smoothing and 10% threshold after every HIO iteration (with exceptions described below). Peak switching was applied after every fifth iteration, with an initial value of β = 1 in equations (2) and (3), reduced to 0.8, 0.6, 0.5 and 0.4, respectively, after every 200 iterations.

We consider three reconstruction cases, each of which was repeated with 25 random initializations. In the first case, no alien was present and the five peaks were phased using CPR with static coupling. In the second case, the alien was added and then manually removed from the 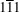 peak prior to phasing by setting a rectangular volume of 92 × 256 × 84 voxels to zero (prior to resampling). Again, CPR was applied with static coupling. In the third case, the alien was added to the 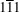 peak and the crystal was phased using the adaptive coupling techniques described in this article. No significant difference was observed in the computational speeds of these three groups.

**Figure 5:**
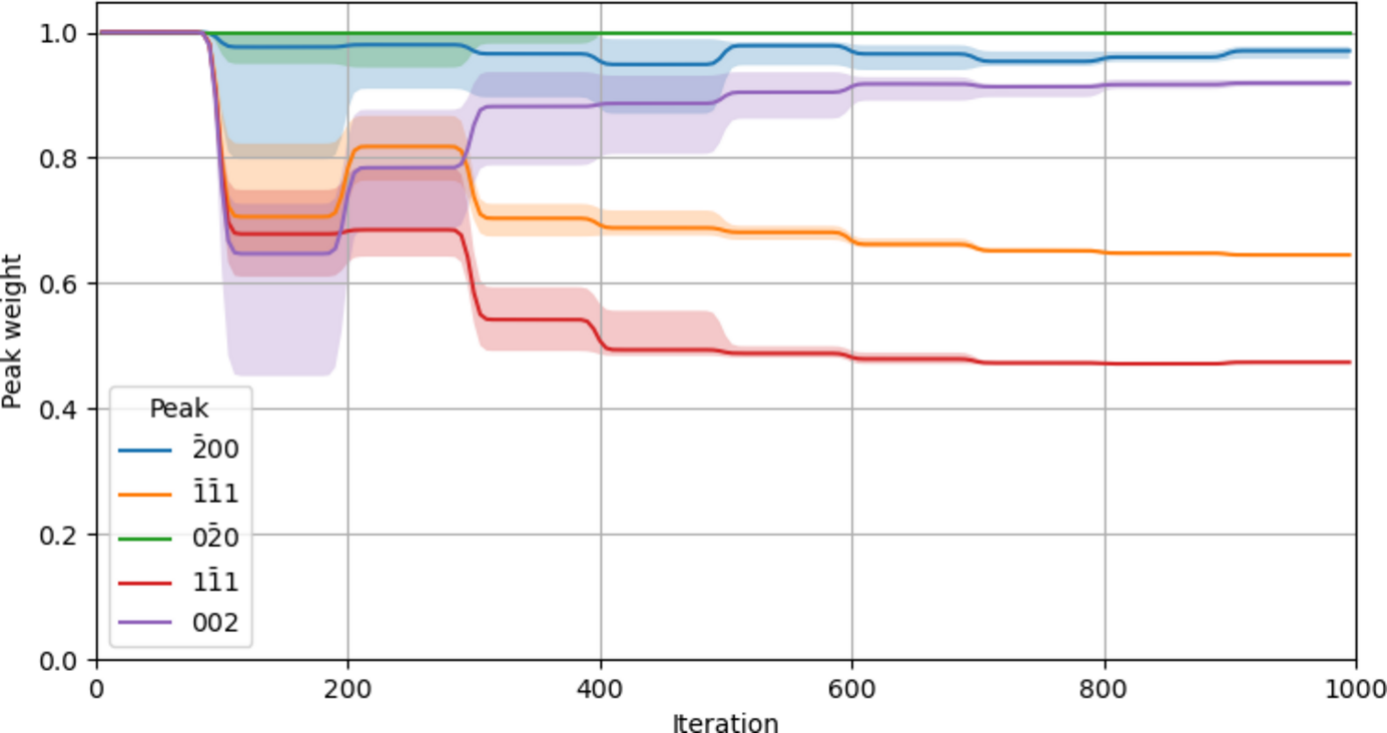
Adaptive coupling weights assigned to each peak over the course of 25 reconstructions. Solid lines indicate median values, while lighter filled regions show the 25th to 75th percentile range. While early weights differ greatly from one reconstruction to the next, the values converge strongly by the end. As expected, the 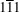 peak (which contained the alien) was given the least weight.

In the adaptive reconstructions (also implemented in *Cohere*), peak weights were initialized at unity and updated every 100 iterations, as shown in Fig. 5 ▸. Despite significant variability in the first few updates, the final weight assigned to each peak was highly consistent across the 25 reconstructions. As expected, the lowest weight was assigned to the alien-containing 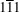 peak. The 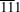 peak was also given a relatively low weight, which could be due to its reduced measurement volume compared with the others. The minimum weight required to apply *Shrinkwrap* to the shared support region while phasing was initially set to *w*_0_ = 0.5 and increased by 0.1 every 200 iterations to a final value of *w*_0_ = 0.9. The threshold for alien detection and removal was fixed at *X*_0_ = 5.

**Figure 6:**
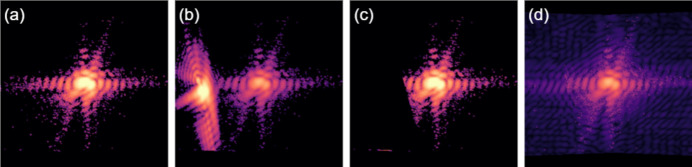
A 2D slice of the Au crystal’s 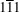 Bragg peak amplitude profile resampled in the crystal reference frame and colored on a logarithmic scale, (*a*) as measured without an alien, (*b*) with an artificially added alien, (*c*) with the alien removed by manual editing and (*d*) with the alien removed by adaptive coupling. The irregular shape of the alien does not fit cleanly into the (originally) rectangular region cut out of panel (*c*), which erases some desired signal and leaves some alien signal. By contrast, adaptive coupling is able to mask a more tightly fitted region around the alien and replaces the erased intensity with projected values. The 3D image arrays were sliced through the center of the alien rather than the main peak.

**Figure 7:**
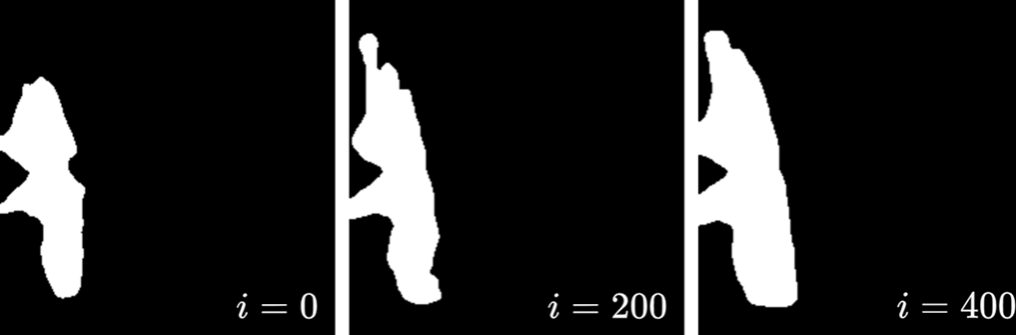
A 2D slice (same index as Fig. 6) of the alien detection mask for the 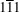 Bragg peak after 0, 200 and 400 iterations of phase retrieval. The mask, initially covering only the brightest parts of the alien, grows as the reconstruction reaches a consensus on the merits of each measured voxel. In these tests, the mask typically stabilized after approximately 400 iterations and showed little change thereafter.

**Figure 8:**
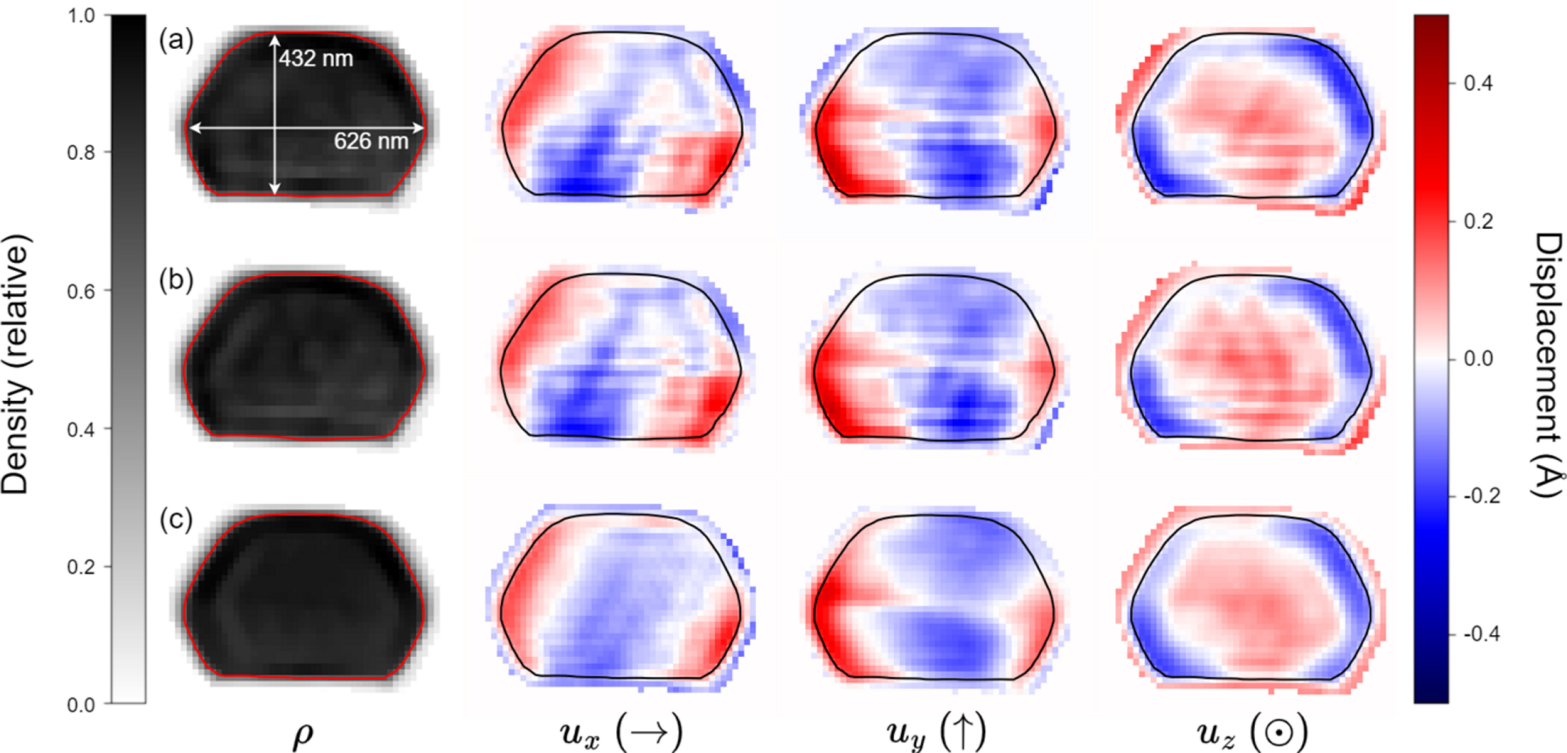
Table of cross-sectional images, showing the atomic density and displacement as reconstructed from five Bragg peaks using (*a*) static coupling with no alien present, (*b*) static coupling with an alien manually removed from one peak and (*c*) adaptive coupling with an alien present in one peak. An outline of the 60% density isosurface is overlaid on each image. Each displacement component is labeled, along with an arrow indicating the positive direction, and the size of the cross section is overlaid on the density of panel (*a*). The atomic displacement fields in rows (*a*) and (*b*) show strong oscillations normal to crystal facets – a common Fourier artifact, which is significantly less pronounced in panel (*c*). To see where the cross section is located in the 3D object, see Fig. 4.

Fig. 6 ▸ illustrates how adaptive coupling not only removes the alien but also fills in the space it had occupied. By contrast, manual data editing introduces a large region of zero intensity which, while probably better than the alien, still constrains the reconstruction with information known to be false. Fig. 7 ▸ shows how the alien-detecting part of *Q*_mask_, initially conservative, grows over the course of the reconstruction. Fig. 8 ▸ shows that static coupling also produces oscillations that radiate inward from each facet (Gibbs phenomena) in the reconstructed density and displacement fields. These artifacts are much less pronounced in reconstructions using adaptive coupling.

We may obtain an estimate of resolution by examining the sharpness of reconstructed surfaces, similar to the ‘knife-edge test’ commonly done in 2D imaging with binary-resolution test patterns. In the present case, the density of the actual Au crystal is effectively binary – uniform within and dropping sharply to zero at the surface. The sharpness of the reconstructed density may therefore be treated as a true, if incomplete, accuracy metric. The knife-edge resolution was calculated by taking the median distance between each point in the 25% (outer) density isosurface and the nearest point in the 75% (inner) density isosurface. The median knife-edge widths are given in Table 2 ▸, along with the narrowest and widest (*i.e.* sharpest and blurriest) values in each group.

To test adaptive coupling further, we performed reconstructions on simulated data. The density profile of the simulated crystal was binary, convex and randomly faceted, and each component of the displacement field was generated from random noise passed through a Gaussian filter. Because the crystal was generated in the laboratory frame, its generated Bragg peaks were resampled to simulate different detector positions. While the resampled arrays were 200 × 256 × 256 voxels to simulate 200 angular positions on a 256 × 256 pixel detector, the crystal and its diffraction peaks were initially generated at the higher resolution of 512 × 512 × 512 to prevent aliasing.

Five diffraction patterns were generated, representing the 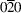, 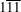, 002, 200 and 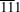 reflections. Aliens generated from other simulated crystals were superimposed onto the 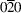 and 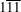 peaks. The 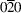 alien was very close to the center of its image, while the 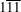 alien was nearer to the edge. Both aliens were adjusted to have 10% of the maximum intensity of their main peak. Two sets of reconstructions were performed: the first using only the 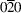, 200 and 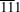 peaks, and the second using all five peaks.

**Figure 9:**
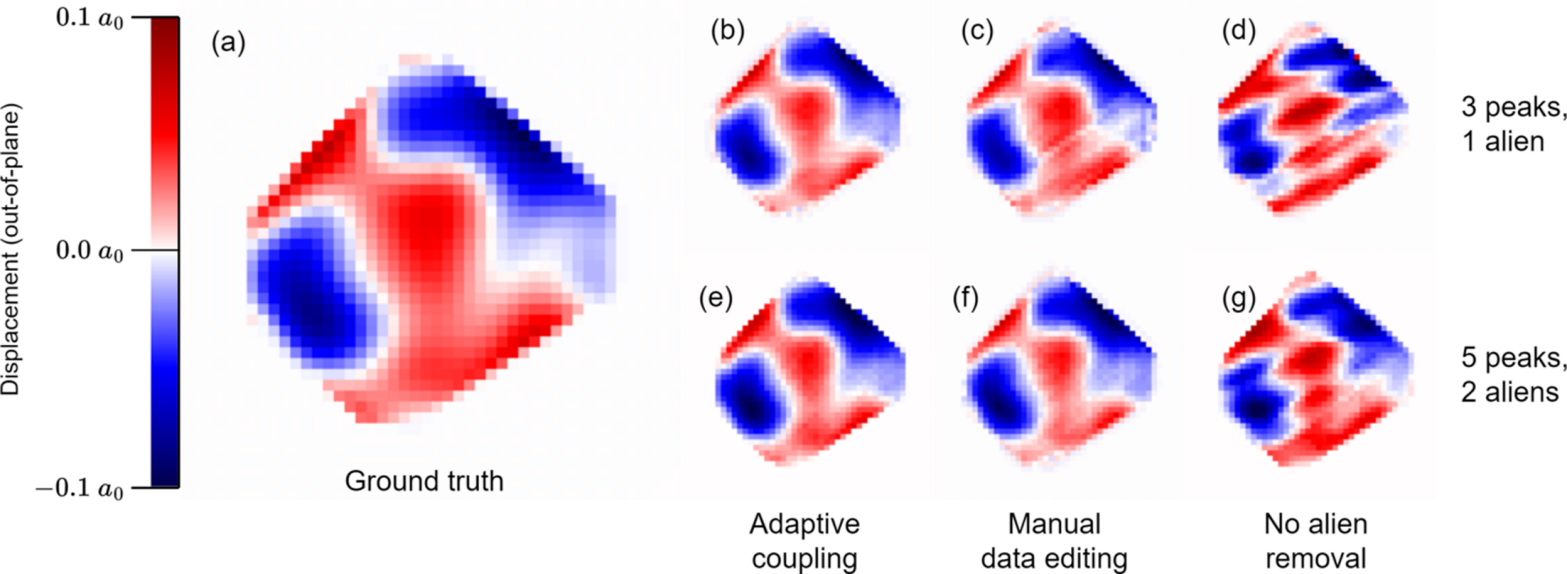
Central cross sections of the out-of-plane displacement field in a simulated crystal, (*a*) downsampled from the original ground truth to match the sampling rate used for phasing, (*b*)–(*d*) reconstructed from three peaks with one alien and (*e*)–(*g*) reconstructed from five peaks with two aliens. Three different reconstruction approaches were used: (*b*) and (*e*) adaptive coupling, (*c*) and (*f*) manual data editing, and (*d*) and (*g*) no alien removal.

The reconstruction parameters for both sets were the same as for the Au nanocrystal, with two exceptions: when sampling back into the detector frame, the diffraction patterns were sampled at 180 × 180 × 180 voxels and the total number of phasing iterations was reduced from 1000 to 500. Each dataset was reconstructed 50 times using adaptive coupling, 50 using manual data editing and 50 with the aliens simply left in place. Fig. 9 ▸ shows a cross section of the displacement field as reconstructed by each of these groups.

**Figure 10:**
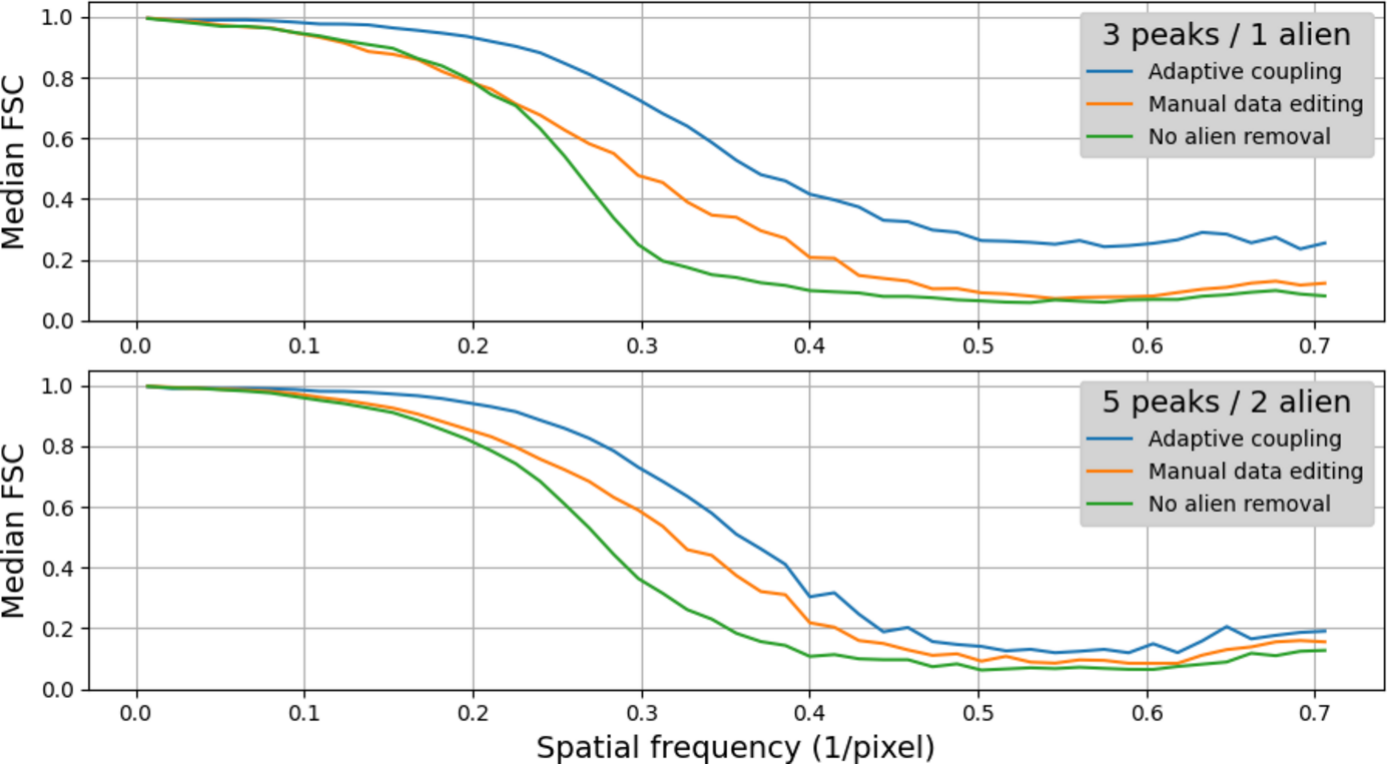
Fourier shell correlations (FSCs) between the reconstructed and true density/displacement fields for various methods and datasets. Each line represents the median FSC across 50 reconstructions. Higher values represent better correlation with ground truth. By this metric, adaptive coupling leads to more accurate reconstruction than manual data editing by a significant margin across the entire range of spatial frequencies.

Each reconstructed crystal was centered, flipped (if a twin image) and projected onto each of the three principle axes via equation (1). A Fourier shell correlation was then used to compare the resulting diffraction images with corresponding projections of the originally simulated crystal. Fig. 10 ▸ gives the median correlation over all three axes and all 50 reconstructions performed per dataset per method. In this test, reconstructions produced with adaptive coupling were significantly more correlated to the ground truth than those produced with manual data editing.

## 4. Conclusion

In this work, we have introduced two complementary adaptive coupling strategies – adaptive peak weighting and adaptive artifact removal – within a CPR framework for multi-peak BCDI. By dynamically adjusting the influence of each Bragg reflection according to its agreement with redundant measurements and by selectively overwriting unreliable voxels, these techniques substantially enhance reconstruction robustness in the presence of imperfect data. This added robustness reduces the need for extensive manual preprocessing of data and allows for rapid reconstructions using pre-transformed diffraction patterns. As a result, adaptive coupling could help beamlines perform high-throughput experiments and enable near-real-time analysis of multi-peak BCDI experiments.

## Supplementary Material

Bragg diffraction measurements for the reconstructed Au crystal shown in the paper.: https://doi.org/10.5281/zenodo.17503854

## Figures and Tables

**Table 1:** Symbols for mathematical notation used herein, with definitions

Symbol	Meaning
*p*	Index signifying a particular Bragg peak
**G**_*p*_	Scattering vector associated with peak *p*
**r**	Location in direct space
**q**	Location in reciprocal space (relative to a given **G**_*p*_)
*I*_*p*_	Measured diffraction intensity for peak *p*
*M*_*p*_	Measured diffraction amplitude for peak *p*
ρ	Electron density within the scattering crystal
**u**	Atomic displacement field within the scattering crystal
ψ_*p*_, Ψ_*p*_	Direct- and reciprocal-space reflections for peak *p* as calculated from ρ and **u**
ψ, Ψ	Direct- and reciprocal-space reflections *as they are being phased*
β	Global update strength
NMI(*A*, *B*)	Normalized mutual information for two arrays *A* and *B*
*H*(*A*)	Shannon entropy of array *A*
*H*(*A*, *B*)	Joint Shannon entropy of two arrays *A* and *B*
*C*_*p*_	List of values returned by equation (4) for peak *p* during the current reconstruction
*w*_*p*_	Weight assigned to peak *p*
*w*_0_	Minimum weight for a peak to apply *Shrinkwrap* software (user defined)
*X*_0_	Threshold for alien detection (user defined)
*Q*_all_	Set of all voxels for which at least one peak has data

**Table 2:** Half-pitch resolution for the sharpest, median and blurriest density reconstructions in each set, estimated by the median distance between the 25% and 75% density isosurfaces Because the transition from diffracting crystal to non-diffracting air is abrupt, this metric partially indicates the accuracy of a reconstruction. According to these tests, adaptive coupling produces sharper reconstructions than manual alien removal.

Method	Narrowest	Median	Widest
No alien	21.8 nm	22.9 nm	23.7 nm
Manual removal	24.7 nm	26.0 nm	26.6 nm
Adaptive coupling	23.1 nm	23.8 nm	24.4 nm

## Data Availability

Data underlying the results presented in this paper are available as supporting information to this article via Zenodo at https://doi.org/10.5281/zenodo.17503854.

## Appendix A Entropy and normalized mutual information

The great insight underlying most of information theory is that the entropy of a distribution – its randomness or unpredictability – is intrinsically related to the amount of information gained by sampling it (Shannon, 1948 ▸). This is why, before planning a picnic for tomorrow, one checks to see whether it will rain but not whether the sun will come up. When discussing the information content of an array *A*, we assume that each element of *A* has been drawn randomly from an unknown (and in the present case, continuous) probability distribution *p*. Fortunately for our numerical machines, *p* can be simultaneously estimated and discretized by taking the histogram of *A*. The Shannon entropy of *A* can then be defined in the usual way,

where  is the set of bins used for the histogram, *a* is an element whose value falls within a given bin and *p*(*a*) is the count for each bin divided by the total number of elements in *A*. Similarly, a joint (2D) histogram can be used to calculate the joint entropy of two arrays *A* and *B*,

where *p*(*a*, *b*) is the probability of finding an *a*-valued element in *A* at the same index as a *b*-valued element in *B*. When calculating these histograms during phasing, the amplitudes in each image were divided into 100 log-spaced bins.

Entropy plays a key role in equation (4), where the similarity between the measured and calculated diffraction amplitudes is expressed using normalized mutual information. In its original form (Studholme *et al.*, 1999 ▸), the NMI between two arrays *A* and *B* is given as

By this definition, perfectly uncorrelated images have an NMI of 1, while perfectly correlated images have an NMI of 2. However, many implementations (including our own) shift this to the more intuitive range [0, 1] by subtracting unity (Feixas *et al.*, 2014 ▸). In either case, however, the NMI is large when knowing the value of some element in *A* allows one to reliably predict the value of the corresponding element in *B*. As might be expected, an image has maximum NMI with a perfect copy of itself. Less intuitively, adding a constant offset to an image does not affect its NMI with any other image. In fact, NMI is insensitive to any operation that maps similar ranges to other similar ranges [*i.e.*]. For this reason, it was developed for use in aligning biomedical images, where two images of the same tissue, taken using different modalities, might appear drastically different yet have the same underlying structure (Studholme *et al.*, 1999 ▸). In the present context this is a desirable feature, since it allows us to compare images that may have different actual intensities, and it does not favor high-intensity over low-intensity regions.
